# The Hospital for Women

**Published:** 1883-09

**Authors:** 


### The Hospital for Women.

We notice with pleasure the establishment in this city of a private hospital or sanitarium for women, as we believe the institution will supply a much-needed want. It is a well-known fact that there are many objections to the care of gynecological cases in large public hospitals, and the dangers to patients, and the obstacles to their recovery, are greatly lessened in smaller and home-like hospitals, which are conducted with peculiar care as to the welfare and comforts of their inmates. From personal observation, we can heartily commend the new hospital.

The building occupied is not only well fitted for its purposes, but is furnished with every comfort for patients, and located in the most desirable part of the city. The charges, too, are extremely moderate, and in view of the advantages it offers in promoting the more speedy and certain restoration to health, it is to be commended to patients even on economic grounds, in preference to the large or general hospital. The well-established reputation of the gentlemen who have organized and placed in working order the gynecological hospital, is a guarantee of good faith and skillful service, and will entitle the institution to the confidence of both the profession and the public. We refer our readers to the advertisement of the Women's Hospital in this number of the JOURNAL for full particulars as to to terms, etc.

